# Glioma grading by microvascular permeability parameters derived from dynamic contrast-enhanced MRI and intratumoral susceptibility signal on susceptibility weighted imaging

**DOI:** 10.1186/s40644-015-0039-z

**Published:** 2015-03-21

**Authors:** Xiaoguang Li, Yongshan Zhu, Houyi Kang, Yulong Zhang, Huaping Liang, Sumei Wang, Weiguo Zhang

**Affiliations:** Department of Radiology, Institute of Surgery Research, Daping Hospital, Third Military Medical University, Chongqing, 400042 China; Department of Radiology, Tianchang people’s hospital, Tianchang, Anhui 239300 China; State key laboratory of Trauma, Burns and Combined Injury, Institute of Surgery Research, Daping Hospital, Third Military Medical University, No. 10, Changjiang Zhilu, Da Ping, Yuzhong Distriction, Chongqing, 400042 China; Division of Neuroradiology, Department of Radiology, Hospital of the University of Pennsylvania, Philadelphia, PA19104 USA

**Keywords:** Brain tumor, Glioma, Grading, Dynamic contrast-enhanced MRI, Susceptibility weighted imaging, Intratumoral susceptibility signal

## Abstract

**Background:**

Dynamic contrast-enhanced MRI (DCE-MRI) estimates vascular permeability of brain tumors, and susceptibility-weighted imaging (SWI) may demonstrate tumor vascularity by intratumoral susceptibility signals (ITSS). This study assessed volume transfer constant (K^trans^) accuracy, the volume of extravascular extracellular space (EES) per unit volume of tissue (V_e_) derived from DCE-MRI, and the degree of ITSS in glioma grading.

**Methods:**

Thirty-two patients with different glioma grades were enrolled in this retrospective study. Patients underwent DCE-MRI and non-contrast enhanced SWI by three-tesla scanning. K^trans^ values, V_e_, and the degree of ITSS in glioma were compared. Receiver operating characteristic (ROC) curve analysis determined diagnostic performances of K^trans^ and V_e_ in glioma grading, and Spearman’s correlation analysis determined the associations between K^trans^, V_e_, ITSS, and tumor grade.

**Results:**

K^trans^ and V_e_ values were significantly different between low grade gliomas (LGGs) and both high grade gliomas (HGGs) and grade II, III and IV gliomas (*P* < 0.01). The degree of ITSS of LGGs was lower than HGGs (*P* < 0.01), and the ITSS of grade II gliomas was lower than grade III or IV gliomas. K^trans^ and V_e_ were correlated with glioma grade (*P* < 0.01), while ITSS was moderately correlated (*P* < 0.01). K^trans^ values were moderately correlated with ITSS in the same segments (*P* < 0.01).

**Conclusion:**

K^trans^ and V_e_ values, and ITSS helped distinguish the differences between LGGs and HGGs and between grade II, III and IV gliomas. There was a moderate correlation between K^trans^ and ITSS in the same tumor segments.

## Background

The angiogenesis of intracranial gliomas plays an important role in evaluating the biological activity and malignancy of a tumor. Tumor vascularity is mostly immature neovascularity consisting of endothelial cells and basement membranes with incomplete structures, resulting in an increase in microvascular permeability. The degree of this increase is associated with tumor type and the degree of malignancy. Moreover, angiogenesis are prone to bleeding, and advanced tumors are inclined to have more angiogenesis and the increased formation of micro-hemorrhage [1-3]. Currently, DCE-MRI may provide information about neovascularity and angiogenesis in gliomas mainly through two important quantitative parameters, K^trans^ and V_e_ [4,5]. K^trans^ is the volume transfer constant in unit time for the transfer of contrast medium from the vessel into the EES, which reflects the intratumoral microvascular permeability. V_e_ is the volume fraction of contrast medium leaking into the EES. SWI is extremely sensitive to the vascular structures and blood metabolites. Researchers have found that parameters associated with DCE-MRI and the degree and distribution of ITSS are significantly correlated with the grades of gliomas [6-10]. These two methods can reveal the pathophysiological state of glioma microvessels from different angles. Therefore, in the present study, it was inferred that a large number of angiogenesis with imperfect functions may reside within the ITSS regions and that ITSS grades may excellently correspond with the maximal K^trans^ value, so these two parameters were both applied to diagnose glioma grades. In the present study, these two methods were applied to assess gliomas, to evaluate the accuracy and value of the associated parameters in diagnosing the grades of gliomas, and to analyze the correlation between the K^trans^ value and ITSS in the same tumor section as well as the relations between these two parameters and microvessel density(MVD) and vessel diameter(VD).

## Methods

### Patient selection and histopathological diagnosis

This retrospective study was approved by the institutional review board of our hospital group. All patients were scanned for preoperative assessment, and informed consent was obtained from each patient. MR examinations of 32 patients (17 female and 15 male, aged 12-69 years old, mean age 42.6 ± 14.3 years old), including 15 patients with LGGs (7 astrocytomas, 6 oligodendrogliomas, and 2 oligoastrocytomas) and 17 patients with HGGs (3 anaplastic astrocytomas, 3 anaplastic oligodendrogliomas, 2 anaplastic oligoastrocytomas, and 9 glioblastomas), were reviewed. All patients underwent conventional MRI, DCE-MRI, and SWI before surgical resection. The pathologic specimens were classified using the 2007 World Health Organization classification criteria for glioma after craniotomy and tumor total resection [11].

### Imaging protocol

All MR imaging was performed using a 3.0 T MR system (Magnetom Verio, Siemens Medical Solutions, Erlangen, Germany) with an 8-element head matrix coil. The conventional MRI included axial and sagittal T1-weighted, T2-weighted, and axial fluid-attenuated inversion recovery (FLAIR) sequences.

DCE-MRI was performed using the sequences described below. First, a baseline T1-weighted MRI (TR/TE = 5.08/1.74 ms, FOV = 260 mm × 260 mm, matrix = 138 × 192, slice-thickness = 5 mm, and flip-angles of 2° and 15°) was used to create two precontrast datasets. Then, a DCE perfusion imaging dynamic series was performed using a T1-twist sequence with a flip angle of 12° (TR/TE = 4.82/1.88 ms, FOV = 260 mm × 260 mm, matrix = 138 × 192, slice thickness = 3.6 mm), which was comprised of 70 measurements with a temporal spacing of approximately 8 s. At the beginning of the baseline acquisition, a bolus of 0.1 mmol/kg gadolinium (Gd)-DTPA contrast agent (Omniscan, GE Healthcare, Shanghai, China) was injected intravenously at a rate of 4 ml/s.

SWI was performed using a 3D fully flow-compensated gradient-echo sequence, and the detailed parameters were as follows: TR/TE = 28.0/20.0 ms, flip angle = 15°, FOV = 230 mm × 230 mm, FOV phase = 75%, SNR = 1.00, slice thickness = 1.2 mm, total acquisition time = 5 min and 5 s, voxel size = 0.8 × 0.7 × 1.2 mm.

### Image analysis

#### Quantitative analysis of DCE images

K^trans^ and V_e_ values were estimated using Tissue-4D software in a Siemens Syngo MR workplace, which was based on the two-compartment pharmacokinetic model by Tofts and Kermode [12]. K^trans^ and V_e_ measurements were acquired by simultaneous observation of axial post-contrast T1-weighted MRI and corresponding K^trans^ and V_e_ maps. The ROI (region of interest) was selected from the axial post-contrast T1-weighted images and then automatically transformed into the corresponding parametric maps. For each tumor, 3-5 ROIs of 40-60 mm^2^ were manually positioned on the corresponding slices of K^trans^ and V_e_ maps by an experienced radiologist. Selections of ROIs within the tumor zone were continued unless a maximal K^trans^ value inside an ROI was acquired. To avoid necrotic, cystic, and hemorrhagic regions, ROI selection was based on enhanced T1-weighted images.

#### Semi-quantitative analysis of SWI images

The degree of ITSS within tumors included 4 grades according to the methods described in a previous review by Park et al. [10], No ITSS represented grade 0, 1-5 dot-like or fine linear ITSS represented grade I, 6-10 dot-like or fine linear ITSS represented grade II, and ≥ 11 dot-like or fine linear ITSS in the continuous region represented grade III. To observe the corresponding relations between maximal K^trans^ value areas and areas with the most densely prominent ITSS, K^trans^ and SWI images were co-registered using Image Pro Plus 6.0 (Media Cybernetics, Inc. USA).

### Measurements of mean MVD and VD values

The MVD and VD of the surgical specimens immunohistochemically stained with anti-CD34 were evaluated. The measurements of the mean MVD and VD values were attained using a computer-assisted image analysis system (Leica, Olympus, Italy). The counting method described by Weidner et al. [13] was adopted for the evaluation of MVD. Then, at least 5 transversally sectioned vessel sections with a single layer of endothelial cells were chosen for each hotspot with or without the thin basement membrane. The mean VD was calculated from the minimum to the maximum diameter of those given vessel sections.

### Statistical analysis

Statistical analysis was performed using SPSS software (version 18.0, SPSS Inc., Chicago, IL, USA). All data were expressed as the mean ± standard deviation (SD). Analysis of variance (ANOVA) was used to compare the values of K^trans^, V_e_, MVD, and VD. The Kruskal-Wallis test was executed to compare ITSS degrees among different grades of gliomas. The ROC curve analysis was conducted to decide the cut-off value with the diagnostic performance of K^trans^ and V_e_ for glioma grading. Relationships between those parameters such as K^trans^, V_e_, degree of ITSS, tumor grade, MVD, and VD were respectively analyzed using Spearman’s correlation. For all statistical tests, *P* < 0.05 was considered statistically significant.

## Results

### The effectiveness of K^trans^ and V_e_ values in glioma grading

The mean K^trans^ and V_e_ values of LGGs and HGGs are shown in Table 1. It can be seen that the K^trans^ and V_e_ values were significantly higher in HGGs than those in LGGs (*P* < 0.01). The mean K^trans^ and V_e_ values of grade II gliomas were significantly lower than those of grade III or IV gliomas (*P* < 0.01). However, no significant differences in K^trans^ and V_e_ values between grade IV and grade III gliomas were found (Table 2). Both K^trans^ and V_e_ values were strongly correlated with glioma grade (*r* = 0.782 and 0.717, respectively, *P* < 0.01).

**Table 1:** **The mean K** ^**trans**^ **,V**_**e**_ **values,MVD,VD values and ITSS grade of different grades of gliomas**

**Tumor grade**	**K** ^**trans**^ **min** ^**−1**^	**V**_**e**_	**MVD**	**VD(μm)**	**ITSS grade**
LGG(grade II)	0.026 ± 0.019	0.121 ± 0.130	14.75 ± 4.94	5.10 ± 1.08	1.2
grade III	0.096 ± 0.063	0.483 ± 0.225	26.84 ± 9.17	7.93 ± 1.34	2.8
grade IV	0.135 ± 0.068	0.525 ± 0.180	22.79 ± 3.51	9.83 ± 1.43	2.4
HGG(grade III and IV)	0.117 ± 0.066	0.505 ± 0.197	24.70 ± 6.87	8.93 ± 1.66	2.6

NOTE—K^trans^ min^−1^: volume transfer constant, V_e_: volume of extravascular extracellular space (EES) per unit volume of tissue, MVD: microvessel density, VD: vessel diameter.

**Table 2:** **P values from K** ^**trans**^ **, V**_**e**_ **, MVD, VD and ITSS grade for differentiation between different grades**

**Grade**	**K** ^**trans**^	**V**_**e**_	**MVD**	**VD**	**ITSS grade**
II vs III	0.003	0.000	0.000	0.000	0.002
II vs IV	0.000	0.000	0.000	0.000	0.032
III vs IV	0.114	0.618	0.172	0.004	0.897
LGG vs HGG	0.000	0.000	0.000	0.000	0.001

The ROC curve analyses of K^trans^ and V_e_ values between different grades of gliomas are shown in Table 3. The cut-off value of K^trans^ (0.054 min^−1^) for differentiation between LGGs and HGGs provided the best combination of sensitivity (94.1%) and specificity (93.3%), and the area under the curve (AUC) of K^trans^ was 0.941. The cut-off value of V_e_ (0.296) provided the best combination of sensitivity (92.9%) and specificity (91.7%), and the AUC of V_e_ was 0.937. Additionally, the different cut-off values of K^trans^ and V_e_ for differentiation between grade II and grade III or IV gliomas also indicated diagnostic accuracy.

**Table 3:** **ROC curve analyses of K** ^**trans**^ **and V**_**e**_ **values for differentiation between the different grades**

	**Threshold**	**Sensitivity**	**Specificity**	**AUC mm** ^**2**^ **.s** ^**−1**^
K^trans^ min^−1^				
LGG vs HGG	0.054	94.1%	93.3%	0.941 × 10^3^
II vs III	0.045	87.5%	86.7%	0.883 × 10^3^
II vs IV	0.064	100%	93.3%	0.993 × 10^3^
V_e_				
LGG vs HGG	0.296	92.9%	91.7%	0.937 × 10^3^
II vs III	0.296	87.5%	93.3%	0.925 × 10^3^
II vs IV	0.345	88.9%	93.3%	0.948 × 10^3^

### The morphology and degree of ITSS among gliomas

**Figure 1:**
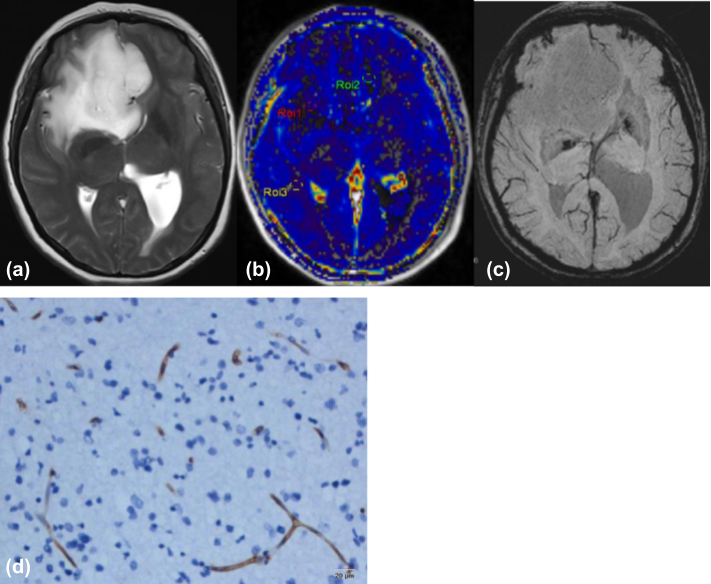
**Images(a-c) of a 37-year-old woman with right frontal low-grade astrocytoma. (a)** Axial T2-weighted image shows an ill-defined mass with high signal intensity. **(b)** K^trans^ map shows low K^trans^ values within the tumor, which is similar to the normal brain tissue. **(c)** SWI demonstrates no evidence of the ITSS. **(d)** Representative immunohistochemical staining(CD34, Original magnification,×200) shows that microvascular hyperplasia is not obvious, which along with low MVD and small VD.

**Figure 2:**
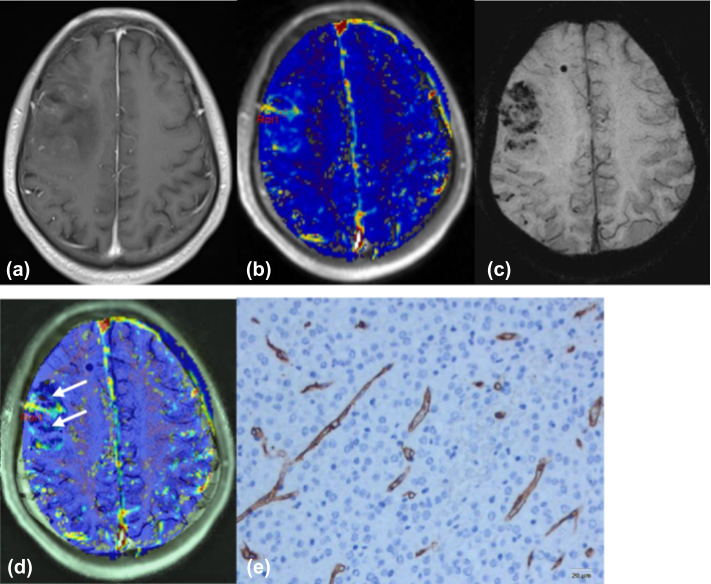
**Images(a-d) of a 26-year-old man with right frontal low-grade Oligodendroglioma. (a)** Contrast-enhanced T1-weighted image shows fair enhancement of the tumor. **(b)** K^trans^ map shows mild increased K^trans^ values within the tumor, relative to the normal brain tissue. **(c)** Multiple dotlike ITSS are shown in the SWI. (d) Coregistered image of K^trans^ and SWI shows that regions of the highest value of K^trans^ does not correspond with areas of attenuated prominent ITSS(arrows) in the same segment. **(e)** Representative immunohistochemical staining(CD34, Original magnification,×200) shows rather abundant mirovessels with small VD.

**Figure 3:**
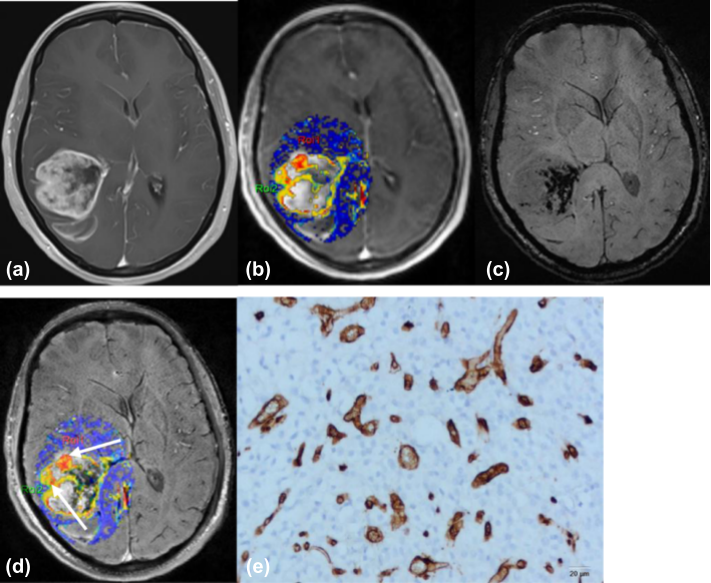
**Images(a-d) of a 44-year-old woman with right temporal anaplastic oligodendrogliomas. (a)** Contrast-enhanced T1-weighted image shows a mass with irregular enhancement. **(b)** K^trans^ map shows high K^trans^ values in the tumor, including **(c)** a maximum degree of ITSS in the SWI. **(d)** Co-registered image of K^trans^ and SWI shows that regions of the highest value of K^trans^(arrows) does not correspond with areas of attenuated prominent ITSS in the same segment. **(e)** Representative immunohistochemical staining(CD34, Original magnification,×200) shows abundant angiogenesis in the tumor, with high MVD, bizarre vascular formation and large VD.

**Figure 4:**
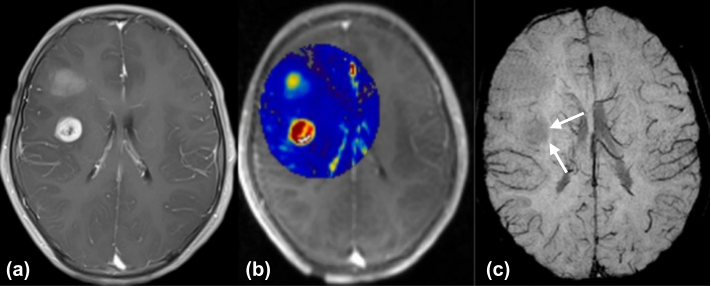
**Images of a 13-year-old boy with left frontal glioblastoma. (a)** The contrast-enhanced axial T1-weighted image shows a mass with regular peripheral rim enhancement. **(b)** The high K^trans^ values within the tumor indicates a high permeability of microvessels. **(c)** However, SWI reveals no evidence of ITSS (arrows).

ITSS were seen in 8 of 9 grade IV patients, in all 8 grade III patients, and in 11 of 15 grade II patients. The Kruskal-Wallis test results showed that the degree of ITSS of LGGs was significantly lower than that of HGGs (*P* < 0.01) (Table 1), and there were significant differences in ITSS degrees between grade II and grade III or IV (*P* < 0.01 and *P* < 0.05, respectively). However, no statistical difference was found between grade III and grade IV gliomas (Table 2). Spearman’s correlation analysis showed a moderate correlation between the degree of ITSS and tumor grade (*r* = 0.515, *P* < 0.01, Table 4). Either no or sporadic dot-like ITSS were found in LGGs (Figure 1c) except for the densely prominent ITSS in the 4 cases of oligodendroglioma (Figure 2c) and single case of oligoastrocytoma. However, the agglomerated mixed nodular and fine linear ITSS were seen frequently in HGGs with the exception of one glioblastoma (Figures 3c and 4c).

**Table 4:** **Correlations between K** ^**trans**^ **values, V**_**e**_ **values, MVD values, VD values, the degree of ITSS and grades by Spearman’s Rho, respectively**

		**K** ^**trans**^	**V**_**e**_	**Grade**	**MVD**	**VD**
K^trans^	Correlation Coefficient	1.000	.823**	.782**	.474**	.692**
	Sig. (2-tailed)	.	.000	.000	.006	.000
V_e_	Correlation Coefficient	.823**	1.000	.717**	.379*	.586**
	Sig. (2-tailed)	.000	.	.000	.032	.000
ITSS	Correlation Coefficient	.473**	-	.515**	.562	.621
	Sig. (2-tailed)	.006	-	.003	. 002	.000

**. Correlation is significant at the 0.01 level (2-tailed).

*. Correlation is significant at the 0.05 level (2-tailed).

### The relationship between K^trans^, V_e_, and the degree of ITSS

K^trans^ values were also strongly correlated with V_e_ values (*r* = 0.823, *P* < 0.01) and moderately correlated with the degree of ITSS (*r* = 0.473, *p* < 0.01) (Table 4). In LGGs, either no or sporadic dot-like ITSS were observed in the SWI images of the 7 cases of astrocytomas, while signs of intratumoral hypoperfusion were observed in the K^trans^ maps. Therefore, these two findings were consistent with each other (Figure 1b-c). Of the 6 cases of oligodendrogliomas, 4 had densely prominent ITSS in their SWI images (1 case of grade 2 and 3 cases of grade 3). However, their intratumoral perfusions in the K^trans^ maps were not too high (the mean K^trans^ value of the 6 cases was 0.043). Thus, in the co-registered image, areas of densely prominent ITSS did not completely correspond to the areas of maximal K^trans^ values (Figure 2d). Moreover, a phenomenon of non-correspondence between the areas of most densely prominent ITSS and the areas of maximal K^trans^ values was found in 1 out of 2 cases of oligoastrocytomas.

In HGGs, the different quantities and morphologies of ITSS were situated at the center or the inner portion of the enhancing rim. In DCE-MRI, the highest value of K^trans^ was located at the areas near the enhancing rim on T1-weighted contrast enhanced images. Co-registered image of K^trans^ and SWI confirmed that the nodular or fine linear ITSS areas partly corresponded to the regions of the highest value of K^trans^ in the same tumor segment (Figure 3d). Remarkably, there was one glioblastoma on SWI without any signs of ITSS which exhibited significantly high vascular permeability (high K^trans^ values) on DCE-MRI (Figure 4b-c).

### The correlation between MVD/VD and K^trans^/V_e_/ITSS

Endoscopic observations indicated that in low grade astrocytomas, their microvascularity was sparse, low in MVD, and small in VD, and their vascular structures were mostly complete (Figure 1e). In oligodendrogliomas or oligoastrocytomas, more angiogenesis with branch-like vessels and a higher MVD were observed, but their VD values were also small. In HGGs, significantly increased in MVD, vessels with irregular and disorderly structures, and enlarged VD were observed (Figure 3e). The mean MVD (14.75) and VD (5.10 μm) of LGGs were significantly lower than those of HGGs (MVD = 24.70, VD = 8.93 μm), and there were significant differences in both the MVD and VD values between grade II and grade III or IV gliomas (*P* < 0.01). No statistical differences in MVD values were found between grade IV and grade III gliomas. However, there were significant differences in VD values between grade IV and grade III gliomas (Tables 1 and 2).

K^trans^ values were moderately correlated with MVD values (*r* = 0.474, *P* < 0.01) but strongly correlated with VD values (*r* = 0.692, *P* < 0.01). V_e_ values were weakly correlated with MVD values (*r* = 0.379, *P* < 0.05) and moderately correlated with VD values (*r* = 0.586, *P* < 0.01). Conversely, ITSS grades were moderately correlated with MVD values (*r* = 0.562, *P* < 0.01) and strongly correlated with VD values (*r* = 0.621, *P* < 0.01) (Table 4).

## Discussion

In gliomas, particularly malignant ones, neovascularity was significantly increased with extremely irregular morphologies and composed of endothelial cells and a basement membrane with incomplete structures. In this neovascularity, vascular resistance significantly increased and intravascular pressure rose, which usually resulted in an increase in vascular permeability and the high likelihood of ruptures and bleeding. To some extent, DCE-MRI and/or SWI reflected that the above pathophysiological changes in tumor neovascularity were somehow associated with glioma malignancy [4,5,8-10]. In low grade gliomas (LGGs), especially astrocytomas, there were no or sporadic dot-like ITSS within tumors, which displayed low K^trans^ values. Thus, there was consistency between ITSS and K^trans^ values, indicating the unchanged permeability and low density in tumor neovascularity, while the vascular characteristics of oligodendrogliomas and oligoastrocytomas were quite different from those of astrocytomas since their grades of ITSS were higher and K^trans^ values were relatively lower. Additionally, a phenomenon of non-correspondence between the ITSS tufts and the areas of maximal K^trans^ values often occurred in oligodendrogliomas and oligoastrocytomas. This phenomenon indicated that vascular structures in the significantly increased tumor neovascularity were nearly complete. Thus, the vascular permeability was not significantly changed, whereas in HGGs, conglomerated dot-like and fine linear ITSS were frequently observed with significantly increased K^trans^ values. Notably, areas of the highest value of K^trans^ did not always accurately correspond to the exact region with the densest ITSS. Considering these radiographic inconsistencies between DCE-MRI and SWI in HGGs, the main reason was hypothesized to be that the detection of ITSS in HGGs not only reflected tumor vascularity distribution but also indicated considerable susceptibility associated with micro-hemorrhage and necrosis within tumors, while the areas of the highest value of K^trans^ represented vascularization with a high proportion of immature, hyperpermeable microvessels.

In this study, K^trans^ values of HGGs were significantly higher than those of LGGs, which meant that HGGs had higher microvascular permeability. So, contrast agents were transferred from plasma to the EES more easily, and plenty of malignant, immature, and hyperpermeable microvessels existed in HGGs. The ROC curve analysis results showed that the cut-off values of K^trans^ provided good diagnostic efficacy (their diagnostic sensitivity and specificity were either near or above 90%) for distinguishing between LGGs and HGGs and between grade II and grade IV, which was consistent with the previous report [14]. Therefore, grading gliomas via the assessment of tumor vascular permeability is highly feasible. Spearman’s correlation analysis showed that the K^trans^ value was strongly correlated with tumor grade (*r* = 0.782, *P* < 0.01), so K^trans^ could be a better biomarker to assess the grading of gliomas. However, there was no statistical difference in K^trans^ between grade III and grade IV. This result was concordant with some reports [8]. This might be attributed to their similar pathological microvascular patterns and because there was abundant microvascular hyperplasia within those two malignant progressive gliomas [15]. The pathological data showed that the mean values of both MVD and VD of LGGs were significantly lower than those of HGGs. This was largely because HGGs were prone to stimulating the secretion of VEGF, which might be the vascular morphogen that formed the abnormally large vessels [16,17]. One of the most important factors affecting the K^trans^ value was blood flow. The increase of MVD and VD values caused the increased leakage of the contrast agent in unit time, which occurred more frequently in HGGs. Spearman’s correlation analysis showed K^trans^ values were moderately correlated with MVD values (*r =* 0.474, *P* < 0.01) and strongly correlated with VD values (*r =* 0.692, *P* < 0.01), indicating that K^trans^ values were more vulnerable to the impacts of VD sizes.

V_e_ is defined as the volume fraction of contrast agent transfer from the vessel into the EES, and many findings on the relationship between V_e_ and glioma grade have been mentioned. The present results, being concordant with previous studies [8,18], showed that the mean V_e_ value in LGGs was significantly lower than that in HGGs, indicating that the leakage volume of contrast agent into EES was greater in HGGs than in LGGs. The ROC curve analysis showed that the cut-off value of V_e_ (0.296) also provided high sensitivity (92.9%) and specificity (91.7%), which helped differentiate LGGs from HGGs. The cut-off value of V_e_ (0.345) also provided the best combination of sensitivity (88.9%) and specificity (93.3%), which helped differentiate grade II from grade IV gliomas. Both AUC were greater than 80%. Spearman’s correlation analysis showed that V_e_ values were strongly correlated with K^trans^ values (*r* = 0.823, *P* < 0.01). Moreover, V_e_ could be expressed mathematically as the ratio of the contrast agent quantity that leaked into the EES to the contrast agent quantity that returned to the plasma space [19], indicating a close relationship between K^trans^ and V_e_ values. V_e_ values were weakly correlated with MVD values (*r* = 0.379, *P* < 0.05) but moderately correlated with VD values (*r* = 0.586, *P* < 0.01). This suggested that VD values within gliomas played an important role in the influence of V_e_ values. Thus, the present study also demonstrated that V_e_ could be a biological marker for glioma grading. EES was easily influenced by some factors such as cell density, necrosis, cystic lesions, and extracellular stroma. A previous study showed that necrotic or cystic regions increased the volume of EES [7], while the area with higher cellularity decreased it. M. Aref et al. [20] demonstrated that extracellular spaces and V_e_ measured by both DCE-MRI and microscopic analysis were statistically similar. Therefore, if the volume of the EES was changed, it resulted in a corresponding change in V_e_. With the rapid growth and metabolism requirements of HGGs, the tumors more easily produced regional cellular hypoxia and necrosis or cystic degeneration, which consequently increased EES volume. An animal experiment also confirmed that both the progression of tumor vascularization and the increase of the EES were closely related with tumor growth [21]. The present results showed that the V_e_ value was strongly correlated with tumor grade (*r* = 0.717, *P* < 0.01), and there were significant differences between LGGs and HGGs and between grade II and grade IV gliomas. This phenomenon could be explained by the larger volume of EES in HGGs due to great physiological and metabolic changes.

Clinically, distinguishing grade II from grade III gliomas is very important because the prognoses for patients with grade II gliomas are significantly better than for patients with grade III gliomas [22]. The present study showed that both K^trans^ and V_e_ values of grade II gliomas were significantly lower than those of grade III gliomas (*P* < 0.01). The cut-off values of K^trans^ = 0.045 min^−1^ and V_e_ = 0.296 were adopted for differentiation between grade II and grade III gliomas, and high sensitivity and specificity (greater than 85%) were achieved. Therefore, these results provided important clinical information for judging the development or progression of grade II to grade III gliomas.

Several studies demonstrated SWI was a promising noninvasive method for differentiating between LGGs and HGGs according to the different frequencies and appearances of ITSS [23-25]. ITSS could simultaneously reflect the intratumoral venous structures and micro-bleeding. The present results indicated that the highest degrees of ITSS were observed in almost all HGGs (except for one case of glioblastoma), suggesting that ITSS can be a potentially helpful sign for the correct diagnosis of HGGs. Also, there was a significant difference in ITSS degree between LGGs and HGGs (P < 0.01) and between grade II and grade III or IV gliomas (*P* < 0.01, *P* < 0.05, respectively). This finding was similar to that described by Park et al. [10]. Thus, the degree of ITSS could be used for grading gliomas. However, the present study also found that the degree of ITSS showed a moderate correlation with glioma grade (*r* = 0.515, *P* < 0.01), which was very different from previous studies. The main reason was assumed to be that the grade II oligodendrogliomas and oligoastrocytomas were also enrolled in this study, while former studies only focused on the differences between high grade and lower grade astrocytomas [9,10]. Significantly increased angiogenesis and highly dense vascularity or mild bleeding were observed in either oligodendrogliomas or oligoastrocytomas, so their grades of ITSS were generally rather high. The present study showed that the degree of ITSS was highly correlated with VD (*r* = 0.629) and moderately correlated with MVD, indicating that, with the exception of angiogenesis and micro-bleeds, the VD size may have hugely impacted magnetic susceptibility to some extent.

At the early stage of gliomas, microvessels are similar to normal brain capillaries. However, in the intermediate stage, they become tortuous, disorganized, and dilated, and in the advanced stage, they change into anarchic and aberrant structures with topographies such as multilayered “glomeruloid tufts,” “garland vessels,” and huge dilated vessels [26]. Remarkably, one glioblastoma showed no evidence of ITSS on SWI, indicating that no micro-hemorrhaging, necrosis, or calcification existed within the tumor. However, this tumor prominently showed high K^trans^ values on DCE-MRI, indicating the high permeability of microvasculature. It was unknown why these microvasculatures were not detected by SWI, though it was speculated that the fine microvasculature within the tumors did not have a great enough susceptibility effect, which was depicted using ITSS. Further studies with larger populations are needed to test this deduction.

Tumor enhancement is the relaxation enhancement caused by pericerebral collections of contrast leakage from the blood-brain barrier (BBB) due to BBB destruction or disintegration. BBB destruction in gliomas may result from the vascular damages caused by tumor formation or the immature endothelium of the tumor neovascularity, as these two factors can both contribute to the contrast leakage and further lead to the enhancement [27,28]. K^trans^ values mainly reflected the permeability of the neovascularity. Therefore, intratumoral distributions of high K^trans^ value areas and contrast collection areas were significantly different, with the former located medially to the tumor enhancement areas and the latter residing inside the tumor. ITSS also reflected the angiogenesis degrees. In low grade astrocytomas, changes and distributions of ITSS were consistent with K^trans^ values. However, in oligodendrogliomas, the degree of ITSS was dense while K^trans^ values were not too high, and the areas of densely prominent ITSS did not completely correspond with the areas of maximal K^trans^ values. Therefore, when analyzing the degrees of tumor enhancement and tumor vascular characteristics to obtain a correct grading assessment, special attention should be paid to the changes in intratumoral vascular characteristics reflected by the various MRI parameters.

This study had some limitations. First, the patient population was small, especially for grade IV tumors, and it was difficult to draw meaningful statistical conclusions from these studies. Second, it was a challenge to assess the voxel-to-voxel correlation between abnormal signals of DCE-MRI and pathological specimens. Third, ITSS on SWI were associated with tumor micro-hemorrhage and necrosis, which could potentially degrade the gradient echo images used for DCE-MRI so that the K^trans^ measurements might be unreliable in the areas of considerable ITSS. Therefore, a larger sample size and more appropriate method should be adopted in future studies to test these results.

## Conclusion

K^trans^ and V_e_ values, and ITSS were capable of differentiating LGGs from HGGs as well as grade II from grade III or IV gliomas. It was the first time that we found a moderate correlation between K^trans^ and ITSS in the same glioma segments.
